# Supplementary material for: Genome-wide CRISPR Screens in T Helper Cells Reveal Pervasive Crosstalk between Activation and Differentiation
Source: Cell. 2019 Feb 7;176(4):882–896.e18. doi: 10.1016/j.cell.2018.11.044 (PMC6370901; doi:10.1016/j.cell.2018.11.044)
Supplement: Data S2. Processed Data from All the Steps of the Analysis, Related to Figure 1 [file mmc2.zip › supplemental data/motif analysis/Pparg_homer/homerResults/motif10.similar.html]

motif10

## Information for motif10

C
G
A
T
G
C
A
T
G
T
A
C
G
A
C
T
A
T
C
G
G
C
T
A
T
C
A
G
C
G
T
A
G
C
T
A
T
G
C
A
  
Reverse Opposite:  

A
C
G
T
C
G
A
T
G
C
A
T
A
G
T
C
C
G
A
T
T
A
G
C
C
T
G
A
C
A
T
G
C
G
T
A
C
G
T
A
  

|  |  |
| --- | --- |
| p-value: | 1e-87 |
| log p-value: | -2.003e+02 |
| Information Content per bp: | 1.655 |
| Number of Target Sequences with motif | 1262.0 |
| Percentage of Target Sequences with motif | 10.08% |
| Number of Background Sequences with motif | 2000.0 |
| Percentage of Background Sequences with motif | 5.58% |
| Average Position of motif in Targets | 101.7 +/- 55.3bp |
| Average Position of motif in Background | 98.6 +/- 63.3bp |
| Strand Bias (log2 ratio + to - strand density) | 0.1 |
| Multiplicity (# of sites on avg that occur together) | 1.05 |
| Motif File: | file (matrix) reverse opposite |

### Similar de novo motifs found

|  |  |  |  |  |  |  |  |
| --- | --- | --- | --- | --- | --- | --- | --- |
| Rank | Match Score | Redundant Motif | P-value | log P-value | % of Targets | % of Background | Motif file |
| 1 | 0.606 | A C G T A C G T A C T G A G T C A C G T A G T C C T G A G A C T | 1e-38 | -88.009635 | 4.42% | 2.43% | motif file (matrix) |
